# LncRNA HAGLROS contribute to papillary thyroid cancer progression by modulating miR-206/HMGA2 expression

**DOI:** 10.18632/aging.205321

**Published:** 2023-12-18

**Authors:** Zhaohui Zeng, Shengtao Tang, Liping Chen, Haiwen Hou, Yuan Liu, Juhui Li

**Affiliations:** 1 Department of Nuclear Medicine, Hunan Provincial People’s Hospital (The First Affiliated Hospital of Hunan Normal University), Changsha 410001, Hunan, China; 2 Chenzhou First People’s Hospital, Beihu, Chenzhou 423000, Hunan, China

**Keywords:** papillary thyroid cancer, HAGLROS, miR-206, HMGA2

## Abstract

Objective: Papillary thyroid cancer (PTC) is one of the most serious diseases of the endocrine system. In view of the limited therapeutic effects of current medical methods, this study starts from the molecular level and looks for potential treatments. The interaction between HAGLROS/miR-206/HMGA2 was studied using multi-omics methods, which provided new ideas and methods for future treatments.

Method: Microarray analysis and R language were used for differential analysis to screening experimental targets of lncRNA, miRNA, and mRNA. qRT-PCR was used to detect RNA expression in tissues and cells. Double luciferase reporter assays analyzed and validated binding relationships between different RNAs. Colony formation, flow cytometry, and transwell assays were used to measure the effect of them on cell proliferation, apoptosis, and migration.

Result: Microarray analysis identified lncRNAs, miRNAs, and mRNAs differentially expressed in PTC and normal cells, and selected lncRNA HAGLROS, miR-206, and mRNA HMGA2 as study subjects. LncRNA HAGLROS and mRNA HMGA2 were highly expressed in PTC cells while miR-206 was lowly expressed in PTC cells. LncRNA HAGLROS/HMGA2 can inhibit apoptosis of PTC cells, promote proliferation and migration, and miR-206 promotes the above process. HAGLROS and HMGA2 were negatively correlated with miR-206. shHAGLROS promoted miR-206 expression, inhibited HMGA2 expression and repressed PTC tumor growth in mice.

Conclusions: HAGLROS promotes the growth of PTC by competitively binding to miR-206 to promote HMGA2 expression.

## INTRODUCTION

Thyroid cancer is the most common malignant tumor of the endocrine organs. Papillary thyroid carcinoma (PTC) accounts for 60~80% of all thyroid malignancies with a rising occurrence worldwide [1]. Similar with the other malignant neoplasms, papillary thyroid cancers are related with specific genetic abnormalities and environmental factors as well [2, 3]. Traditionally, studies focus on the environmental factors, such as ionizing radiation and family history. However, Studies of the pathogenesis on gene level are still not enough. Therefore, it is crucial to find new insights into pathogenesis of PTC and develop a novel therapeutic strategy for PTC treatment.

“MixOmics” package proposes multivariate projection-based methodologies for omics data analysis. “MixOmics” can handle large data sets efficiently and perform dimension reduction to finish the visualization [4, 5]. “MixOmics” has been successfully applied to classify or discriminate sample groups and identify the most discriminant subset of biological features [6]. So in this study, we introduced MixOmics to analyze the genetic pathogenesis of PTC.

LncRNAs are a novel class of transcripts composed of more than 200 nucleotides which were originally considered as a gene transcriptional noise [7]. A recent study has provided evidence that lncRNAs play a pivotal role in various regulatory functions, encompassing oncogenesis, metastasis, apoptosis, and invasion. These findings underscore the pressing need to unravel the underlying mechanisms driving these processes [8]. LncRNA also functions as competing endogenous miRNA as a part of ceRNA network [9]. HAGLROS is lncRNA with a length of 699 bp that has recently been found to promote the malignant progression of gastric cancer cells [10]. Nevertheless, the key role of HAGLROS in other cancer like PTC has not been reported.

MiRNAs are another important class of non-coding (ncRNAs) with endogenous 21-23 nucleotides, which effectively regulate post-transcriptional eukaryotic gene expression. MiRNAs are considered to perform vital part in many biologic processes, such as cellular proliferation, differentiation, maturation and apoptosis [11]. MiR-206 was involved in invasion and metastasis of lung and laryngeal cancer [12]. Studies also demonstrated down-regulation of miR-206 in many other neoplasms [13]. All these studies suggest that miR-206 probably acts as a tumor suppressor in multiple cancers. However, the molecular regulatory mechanisms concerning miR-206 and lncRNA HAGLROS remain obscure but attractive targets for exploration.

High mobility group AT-hook 2 (HMGA2), a non-histone chromosomal protein, belongs to high-mobility group protein family. HMGA2 was poor prognosis in different cancers which was shown to promote cellular proliferation, invasion and migration [14]. Study has revealed that HMGA2 was overexpressed in PTC, suggesting that HMGA2 may promote the malignant progression of PTC [15]. However, the molecular regulatory mechanism of HMGA2 still has some shortcoming.

In this study, we use TCGA data and R software to analyze the potential molecular factors that modulated PTC. We investigated the expression of the discovered RNAs in PTC and their effects, like cell growth, metastasis, and apoptosis on PTC cells. In addition, we conduct further experiment to explore the relationship among HAGLROS, miR-206 and HMGA2. Together, study contributes to the characterization of the molecular mechanisms of PTC progression.

## RESULTS

### Bioinformatics analyses with mixOmics package

**Figure 1:**
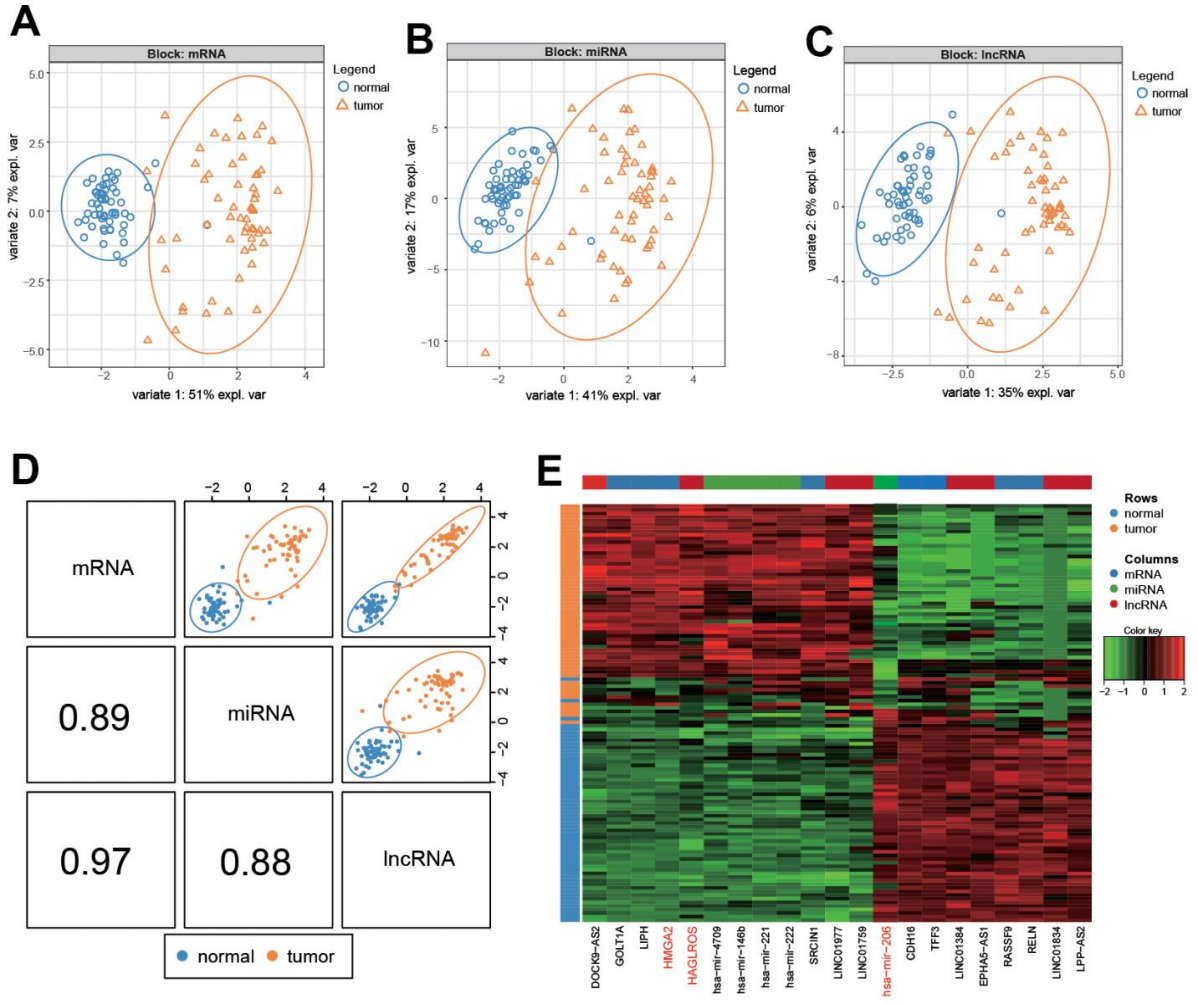
**Differential analysis of RNA in PTC and normal cells.** (**A**–**C**) mRNA, lncRNA and miRNA differentially expressed in PTC and normal tissues. (**D**). Summary of (**A**–**C**). (**E**) Heat map of differential expression of RNA in PTC and normal tissues.

**Figure 2:**
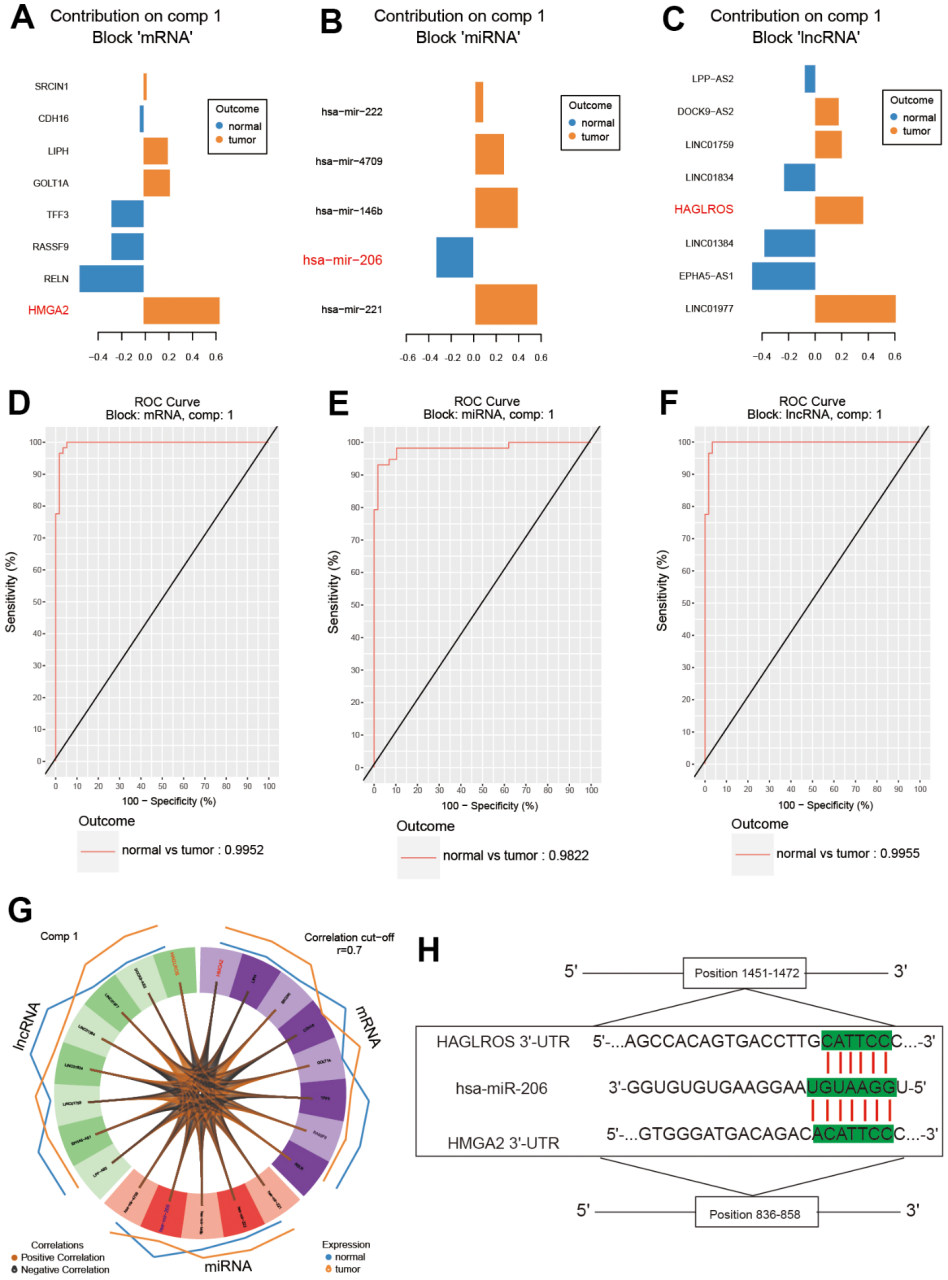
**Analysis of the results of differential expression.** (**A**–**C**) LncRNA HAGLROS, miR-206 and HMGA2 were selected based on differential expression. (**D**–**F**) ROC curves for lncRNA HAGLROS, miR-206 and HMGA2. (**G**) The correlations between differentially expressed RNAs in PTC and normal tissues. (**H**) Possible binding sites among LncRNA HAGLROS, miR-206 and HMGA2.

The results of SPLSDA analysis showed that there was a small amount of separation between PTC tissues and normal tissues in the level analysis of mRNA (Figure 1A) and lncRNA (Figure 1C), and a small amount of overlap between the two groups was found in the miRNA (Figure 1B) analysis (Figure 1A–1C). The correlation plot indicated that the first component was highly correlated to each other (Figure 1D). The above results suggested that genes differentially expressed in PTC and normal tissues (Figure 1E). LncRNA HAGLROS, miR-206 and HMGA2 were selected based on the differences in the three differentially expressed RNAs (Figure 2A–2C). We used receiver operating characteristic curve (ROC) to compare repeated experimental design. The results showed that the AUC (area under the curve) of the first component are all close to 1, indicating the high accuracy of the experiment (Figure 2D–2F). The lateral line of the circos plot indicates the expression level of RNAs, with lower levels being represented by positions further away from the center of the circle (Figure 2G). Binding sequence was found among lncRNA HAGLROS, miR-206 and HMGA2 (Figure 2H).

### Overexpressed lncRNA HAGLROS promoted proliferation and migration while repress apoptosis of PTC cells

**Figure 3:**
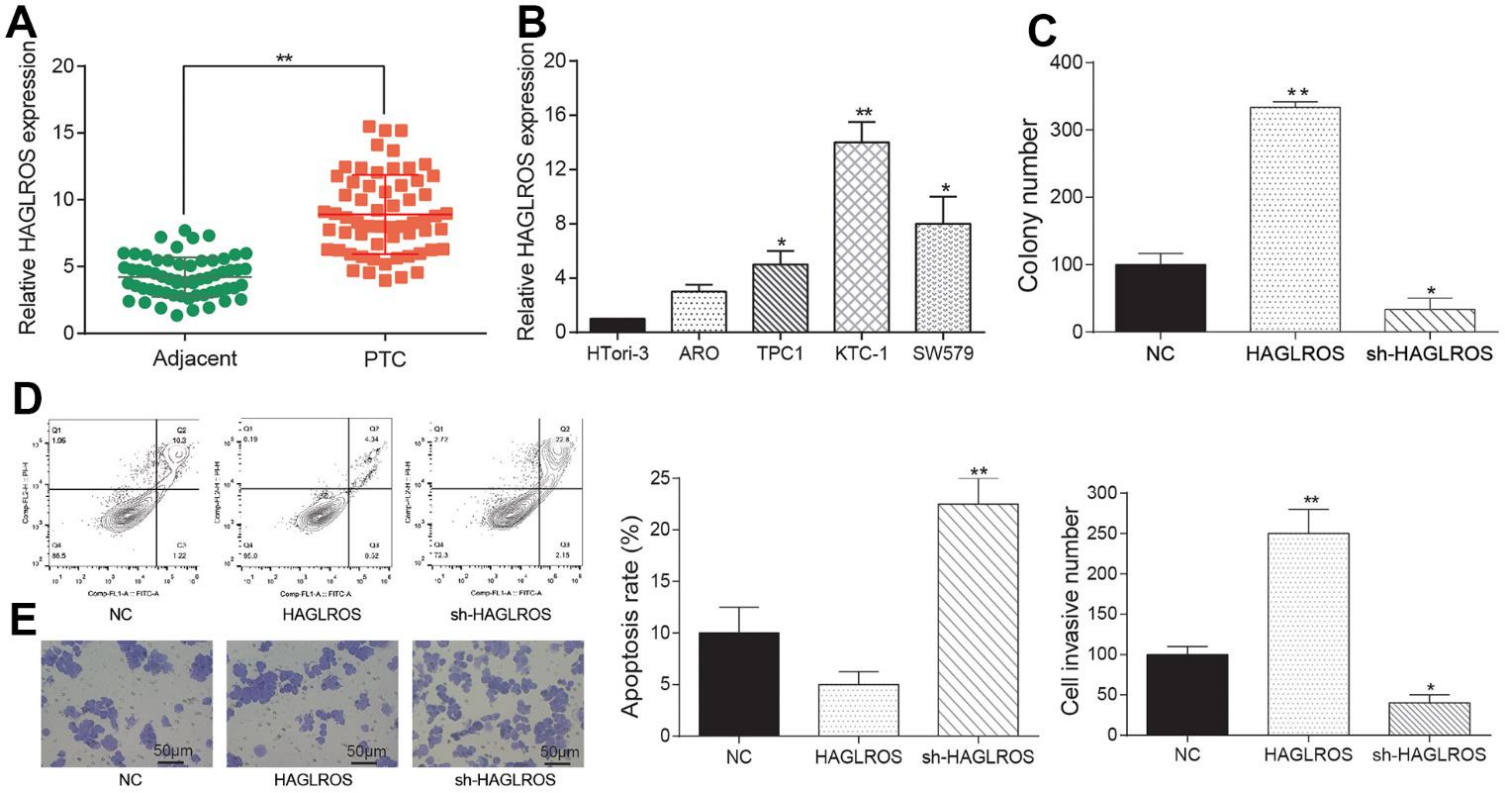
**Overexpressed lncRNA HAGLROS promoted proliferation and migration while repress apoptosis of PTC cells.** (**A**) HAGLROS was highly expressed in PTC tissues. (**B**) The expression of HAGLROS in PTC cells was higher than normal cells. (**C**) HAGLROS promoted PTC cell proliferation. (**D**) HAGLROS inhibited PTC cell apoptosis. (**E**) HAGLROS enhanced cell invasion.

Results of qRT-PCR showed that lncRNA HAGLROS in PTC was upregulated in contrast with adjacent tissues (Figure 3A). The following cell experiments further confirmed this conclusion. Compared with the normal thyroid cell line HTori3, lncRNA HAGLROS was all over-expressed in ARO/TPC1/KTC-1/SW579 PTC cell lines, and the expression level of lncRNA HAGLROS in KTC-1 was the highest (Figure 3B). Taken the above results together, lncRNA HAGLROS may play an important role in PTC. Therefore, we employed KTC-1 cell line to carry out cell experiments of lncRNA HAGLROS *in vitro*. Cell transfection specifically targets HAGLROS and shHAGLROS. Clone formation assay results showed that over-expressed HAGLROS significantly enhanced KTC-1 clonal proliferation capacity compared to NC and shHAGLROS HAGLROS (Figure 3C). Flow-apoptotic results showed that over-expression of lncRNA HAGLROS inhibited KTC-1 apoptosis (Figure 3D). Transwell experiments indicated that up-regulated HAGLROS promoted the invasion of KTC-1 (Figure 3E).

### LncRNA HAGLROS rescues the effects of miR-206 on PTC cells

**Figure 4:**
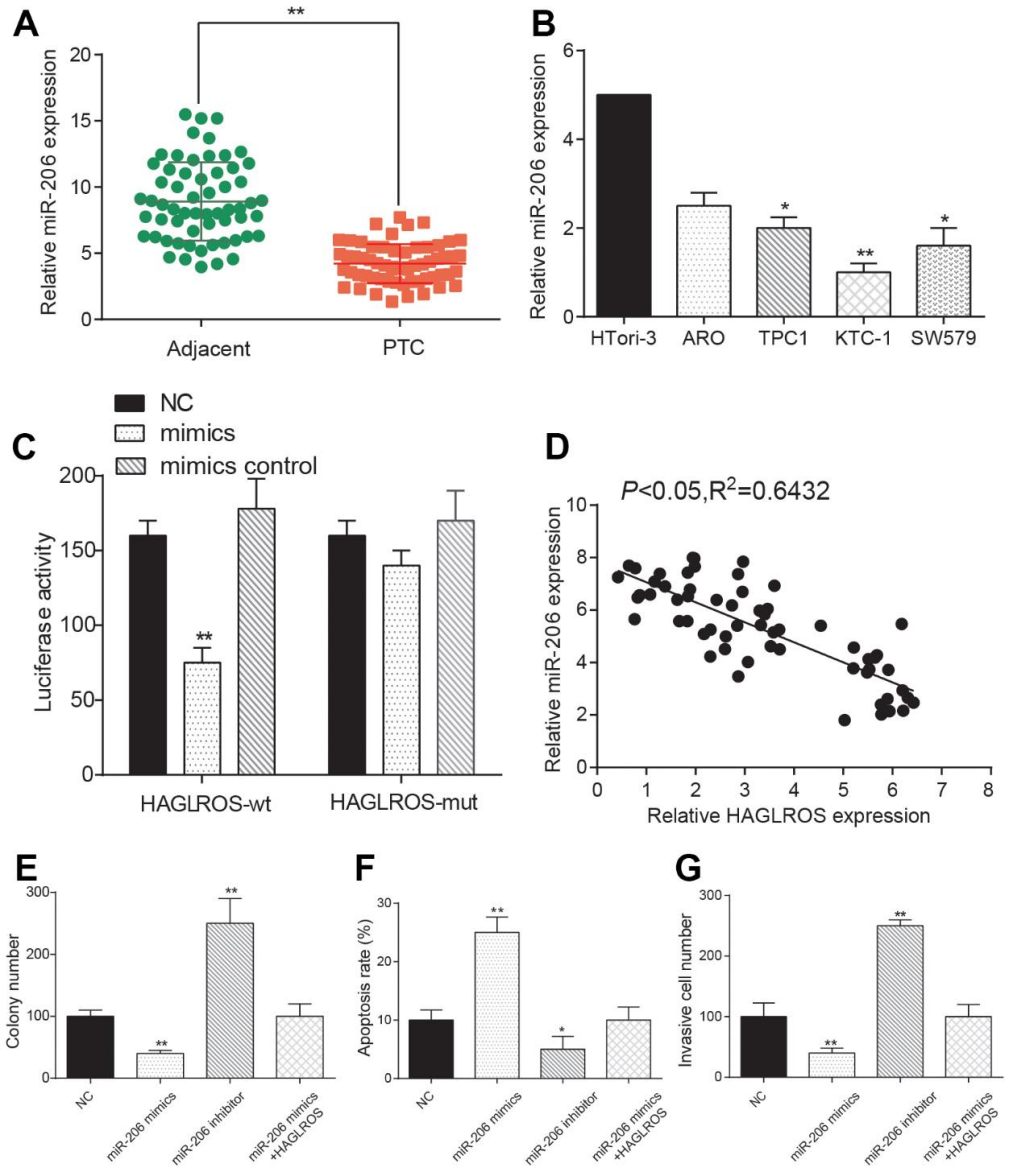
**LncRNA HAGLROS rescues the effects of miR-206 on PTC cells.** (**A**) miR-206 is down-regulated in PTC tissue. (**B**) miR-206 expression in PTC cell lines is lower than normal cells. (**C**) Dual luciferase assay showed that the expression level of miR-206 in HAGLROS-WT was lower than that of HAGLROS-MUT. The NC group was treated with PBS and the mimic control group was treated with nonsense sequence as the control group of mimics. (**D**) Negative correlation between HAGLROS and miR-206. (**E**) miR-206 inhibited proliferation of PTC cells and shHAGLROS+ miR-206 weakened the suppression. (**F**) miR-206 promotes apoptosis of PTC cells and shHAGLROS+ miR-206 weakened the promotion. (**G**) miR-206 inhibits cell invasion and shHAGLROS+ miR-206 weakened the suppression.

The results of qRT-PCR showed that the expression of miR-206 in PTC was lower than that in adjacent normal tissues (Figure 4A), and the results of PTC cell experiment accorded with this (Figure 4B). MiR-206 performed the lowest expression level in KTC-1, so KTC-1 was selected as the cell line for the next experiment. The dual-luciferase reporter system results came out that miR-206 mimic expression in HAGLROS-MUT group was higher than HAGLROS-WT group, indicating that HAGLROS can combine to miR-206 and have an impact on miR-206 expression (Figure 4C). In addition, our RIP data showed that HAGLROS was significantly enriched in the mimics group (Supplementary Figure 1A). As the expression level of HAGLROS increased, the expression level of miR-206 decreased significantly (Figure 4D). The results of colony formation assay, flow cytometry apoptosis assay and transwell assay showed that overexpression of miR-206 inhibited the proliferation and invasion of KTC-1 and promoted the apoptosis of KTC-1. However, when HAGLROS was overexpressed, the inhibitory effect of miR-206 on KTC-1 was reversed, indicating that HAGLROS affected KTC-1 by regulating miR-206 (Figure 4E–4G).

### MiR-206 rescues the effects of HMGA2 on PTC cells

**Figure 5:**
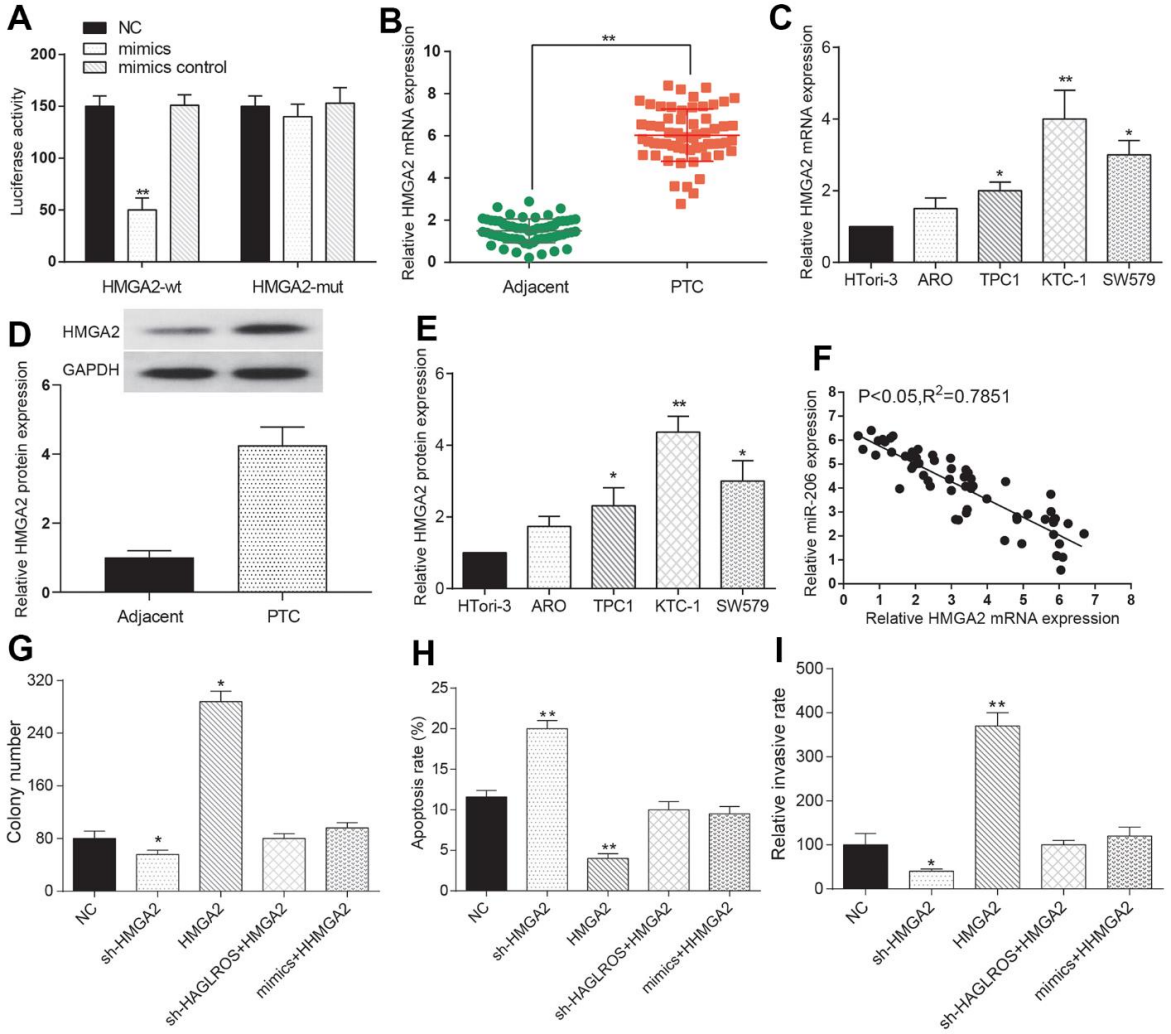
**MiR-206 rescues the effects of HMGA2 on PTC cells.** (**A**) The expression level of miR-206 in HMGA2-WT is lower than that in HMGA2-MUT. The NC group was treated with PBS and the mimic control group was treated with nonsense sequence as the control group of mimics. (**B**) HMGA2 over-expressed in PTC tissue. (**C**) HMGA2 over-expressed in PTC cells. (**D**, **E**) Western blotting was used to detect the protein expression level of HMGA2 in clinical tissues and cells. (**F**) HMGA2 negatively correlated with miR-206. (**G**) HMGA2 promotes PTC cell proliferation and the promotion effect weakened after the addition of shHAGLROS or miR-206. (**H**) HMGA2 inhibited PTC cell apoptosis and effect weakened after shHAGLROS or miR-206 was added. (**I**) HMGA2 enhanced cell invasion ability and the enhancement effect faded after adding shHAGLROS or miR-206.

The dual-luciferase reporter system indicated that the miR-206 mimic expression level in the HMGA2-MUT group was higher compared with that in the HMGA2-WT group, implying that HMGA2 bound to miR-206 and was regulated by miR-206 (Figure 5A). In addition, our RIP data showed that HMGA2 was significantly enriched in the mimics group (Supplementary Figure 1B). The outcome data of qRT-PCR proved that HMGA2 was over-expressed in PTC compared with that in normal adjacent tissues (Figure 5B). The expression of HMGA2 mRNA and protein in PTC cell lines was higher than that in normal cell line HTori3, and the highest expression level came to KTC-1 (Figure 5C–5E). There was a negative correlation between HMGA2 and miR-206 (Figure 5F). Clone formation assay results showed that HMGA2 significantly enhanced cell proliferation and after transfection with shHAGLROS+HMGA2 or miR-206 mimics+HMGA2 the proliferation showed no significant change compared with the NC group (Figure 5G). Flow cytometry experiments showed that HMGA2 could inhibit the apoptosis of KTC-1 and the apoptosis rate after transfection with shHAGLROS+HMGA2 or miR-206 mimics+HMGA2 had no obvious change compared with NC group (Figure 5H). Transwell invasion assay results showed that HMGA2 enhanced the invasion ability of KTC-1 and after transfection with shHAGLROS+HMGA2 or miR-206 mimics+HMGA2 the cell invasion ability has no significant change compared with NC group (Figure 5I). In conclusion, we predicted that HMGA2 could regulate the PTC cell biological behavior under the control of miR-206.

### ShHAGLROS suppressed growth of PTC *in vivo* by modulating miR-206/HMGA2 expression

**Figure 6:**
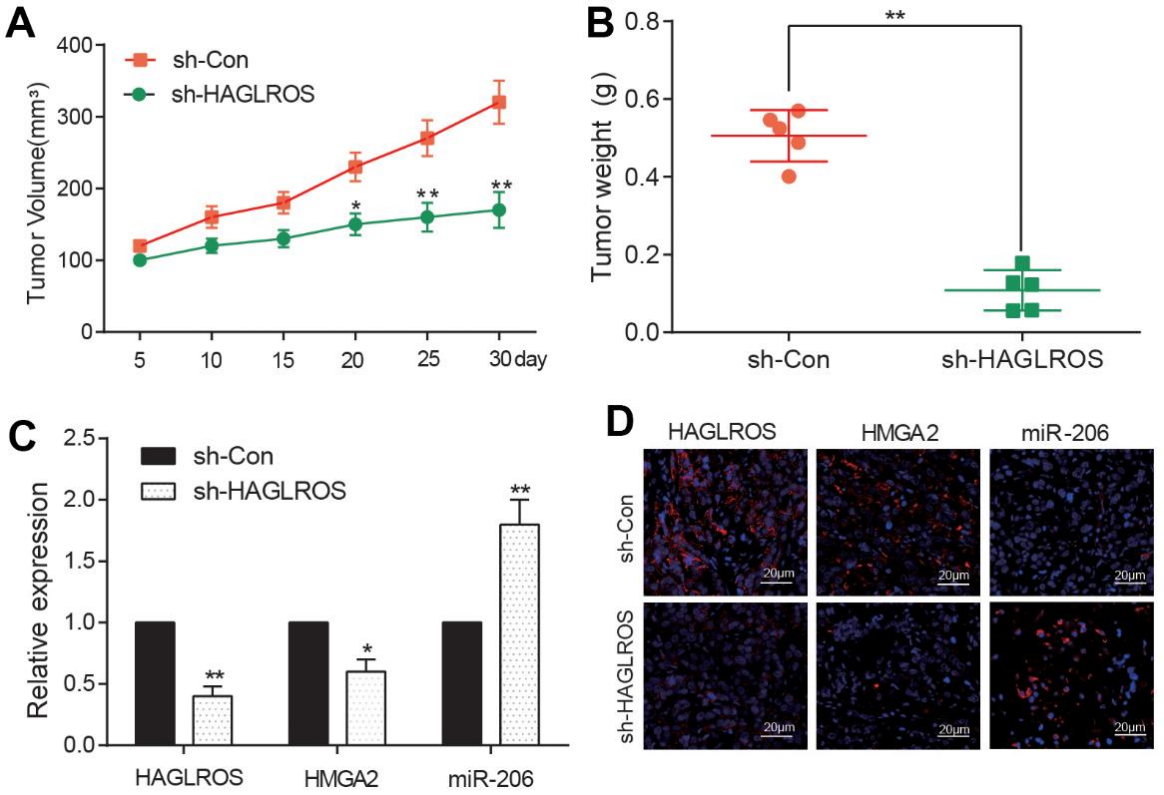
**shHAGLROS inhibits the growth of PTC tumors.** (**A**, **B**) shHAGLROS decreased the volume and weight of PTC tumors. (**C**) The expression of HAGLROS or HMGA2 decreased in PTC tumors in nude mice after addition of shHAGLROS while miR-206 over-expressed. (**D**) The expressions of HAGLROS, HMGA2 and miR-206 in different tumor tissues were detected by immunofluorescence and FISH fluorescence assay.

KTC-1 cells transfected with shHAGLROS and shCon were inoculated into nude mice. The shHAGLROS group also had a lower tumor volume as well as weight compared with the shCon group (Figure 6A, 6B). The measurement of Qrt-PCR revealed that the expression of HAGLROS and HMGA2 in shHAGLROS was obviously lower than that in shCon group. The expression of miR-206 in shHAGLROS group was significantly higher than that in shCon group (Figure 6C). In addition, we also detected the expression of HAGLROS, HMGA2 and miR-206 by immunofluorescence and FISH fluorescence, and found that the expression of HAGLROS and HMGA2 was decreased while the expression of miR-206 was increased in the sh-HAGLROS group (Figure 6D). All the results of experiments *in vivo* correspond with the result *in vitro*, that HAGLROS was shown to promote PTC growth by regulate miR-206 and HMGA2.

## DISCUSSION

In recent years, multi-omics analysis has become more popular in cancer research while traditional single factor analysis cannot reveal the integrated pathogenesis of certain cancer. Many researches demonstrated that lncRNA and miRNA serve functionally as tumor promoting or suppressor genes and regulate multiple cellular processes in oncogenesis. As for the focused PTC, LncRNA AB074169 could suppress the PTC and microRNA-148a has a similar inhabitation on PTC [16, 17]. Based on the ceRNA hypothesis, lncRNAs may act as endogenous decoys for miRNAs to elicit their biological activity so that lncRNA can influence the interaction between miRNAs and their targets [18]. The ceRNA hypothesis inspired us that lncRNA may promote or inhibit carcinoma by regulating certain miRNA. Yang Liu et al. demonstrated that lncRNA HOTTIP promotes cell proliferation, invasion and migration process by modulating miR-637 in papillary thyroid carcinoma [19]. Moreover, studies not focusing on lncRNA also proved miRNA alone to be a tumor suppressor by regulating target mRNA [20]. However, the regulatory mechanism of lncRNA, miRNA and mRNA is not fully understood and based on the previous studies we speculated that certain RNAs might constitute an lncRNA/miRNA/mRNA axis to modulate PTC.

In the present study, we employed mixOmics combined with SPLDA to analyze and selected three kinds of RNA (lncRNA HAGLROS, miR-206, HMGA2) that were differentially expressed in PTC. Furthermore, we transfected HAGLROS into KTC-1 cells to determine the biological function of HAGLROS. We found that lncRNA HAGLROS promoted proliferation, invasion and suppressed apoptosis of PTC cells. Previous studies have shown that HAGLROS promotes the malignant progression of gastric cancer cells, suggesting that HAGLROS may be a malignant factor in cancer [21]. In addition, recent studies have shown that HAGLROS is highly expressed in PTC clinical samples [22]. However, its role and mechanism in PTC are still unclear. This study is the first to explore and verify the mechanism of HAGLROS regulating PTC. On the contrary, we demonstrated that over-expressed miR-206 can be a tumor suppressor to the PTC cells. Recent researches showed that miR-206 was down-regulated in PTC and miR-206 inhibits proliferation and migration process in prostate cancer by targeting CXCL11, which corresponded with our result [23, 24]. Previous studies showed that HMGA2 was associated with multiple cancers. Zhang S et al. proved HMGA2 to be an accelerator of glioma malignance [14]. However, the impact of HMGA2 on PTC has not been deeply probed. Besides the relationship among lncRNA HAGLROS, miR-206 and HMGA2 were still worth to investigated.

In this study, dual luciferase assay confirmed the combination of lncRNA HAGLROS and miR-206. MiR-206 was also proved to be capable of binding with HMGA2 using the same method. The combinations suggested that lncRNA HAGLROS could indirectly influence the expression of HMGA2 via miR-206. Furthermore, we transfected HAGLROS along with HMGA2 into KTC-1 cells, we discovered that effects of HMGA2 on KTC-1 cells was significantly modulated, indicating the existence of lncRNA HAGLROS/miR-206/HMGA2 axis. Earlier studies have described that apoptosis and autophagy of lipopolysaccharides-induced WI-38 cells were modulated by HAGLROS/miR-100/NF-κB axis, which were consistent with our findings [25].

Despite of the exciting findings, some shortcomings in this study cannot be ignored. On the one hand, the sample size from PTC patients is not large enough. On the other hand, the mentioned mechanism is not enough to explain the complex pathogenesis of PTC, while the other lncRNA may also play a important role.

Taken together, our study found HAGLROS/miR-206/HMGA2 axis in PTC and proved that downregulated HAGLROS could suppress PTC. Nowadays, only several studies have explored the lncRNA/miRNA/mRNA axis mechanism in cancer and few to apply to the pathogenesis of PTC. The findings provided a new insight into the diagnostic and therapeutic strategy in the treatment of PTC. And the new lncRNA/miRNA/mRNA axis can also be applied to investigate the unknown mechanism of many other aggressive and therapy resistant cancers.

## MATERIALS AND METHODS

### The cancer genome atlas (TCGA) dataset

The papillary thyroid carcinoma (PTC) datasets were downloaded from the TCGA online database (http://cancergenome.nih.gov/). The raw data of the tumor tissues and corresponding normal tissues was revised and normalized by “DESeq2” and “edgeR” package using R 3.4.1 (https://www.r-project.org/). *P*- value equivalent to 0.05 and log_2_ (Fold Change) equivalent to 1 were the thresholds that used to distinguish the differentially expressed RNAs. Then the differentially expressed RNAs were used for multi-omics analysis with “mixOmics” package.

### Multi-omics analysis by mixOmics package

The R package “mixOmics”, which could fulfill multiple functions such as data mining, dimension reduction and data visualization, was used to conduct multi-omics analysis for biological data sets. According to provider’s instructions (https://www.mixOmics.org), the download data from TCGA were analyzed by Stacked Partial Least-Squares Discriminant Analysis (SPLSDA) in R 3.4.1 software, then the relevance network of first component was finished (r=0.7). Circos plot was output to show expression levels on a circle and strong positive or negative correlations in Omics.

### Cell culture

PTC cell line ARO (BNCC338230), TPC-1 (BNCC337912), KTC-1 (BNCC340144), SW579 (BNCC100182), as well as normal cell line HT-ori3 (BNCC338687) were all purchased from BeNa Culture Collection (Beijing, China). ARO and TPC-1 cells were cultured in 90% RPMI-1640+10% FBS and SW579 in 90% L-15+10% FBS while normal HT-ori3 cells and KTC-1 in 90% F-12K+10%FBS. All cells grew at 95% air+5% CO_2_ condition in a 37° C room. All experimental reagents were obtained from GIBCO (NY, USA).

### Tissue samples collection

Patients diagnosed with PTC who had not undergone local or systemic treatment before the operation were picked up. All the normal surrounding tissue samples and PTC tissue samples used in this study were acquired from the selected patients. All the sample collections obtained from the surgery were immediately quick-frozen in liquid nitrogen, and preserved at −80° C for the following experiments. The research got the permission from the hospital and patients. The clinical information was listed in Table 1.

**Table 1:** Correlation between HAGLROS expression and clinic pathological features in papillary thyroid cancer (PCT) (n=60).

**Characteristics**	**Total**	**High expression**	**Low expression**	**P-value**
Gender				
Male	25	12	13	0.631
Female	35	19	16	
Age(years)				
<45	33	14	19	0.1945
≥45	27	16	11	
Extra thyroidal extension				
Yes	27	13	14	0.6218
No	33	18	15	
TNM staging^[1]^				
I–II	36	13	23	0.0084**
III–IV	24	17	7	
Lymph node metastasis				
Yes	43	20	23	0.6534
No	17	9	8	
Multi centricity				
Yes	36	20	16	0.4603
No	24	11	13	
Tumor size (cm)				
<2	30	11	19	0.0201*
≥2	30	20	10	

*P<0.05.

^[1]^TNM, Tumor Node Metastasis; it’s a standard way for clinicians and medical scientists to stage a malignant tumor.

### Total RNA extraction and qRT-PCR

Total RNA was collected from tissue samples and cells using RNAiso Reagent (Takara, Dalian, China). The qRT-PCR was performed using SYBR Premix Ex Taq II (Takara) on a Light Cycler480 system (Roche, USA). The thermo cycling conditions included an initial denaturation step at 95° C for 30 s, denaturation at 95° C for 5 s, and annealing at 60° C for 30s for 40 cycles, dissociation stage at 95° C for 60 s, 55° C for 1 min, 95° C for 30 s. The primer sequences were shown in Table 2. The 2^−ΔΔCT^ method (CT, cycle threshold) was used to calculate the relative expression levels. GAPDH and U6 were used as reference genes to calculate lncRNA/miRNA/mRNA expression. In this study all RNA expression levels determined by qRT-PCR were relative quantities and not absolute quantities. Each one was performed in triplicate to verify the stability and repeatability of the results.

**Table 2:** qRT-PCR primer.

	**Primer sequences (5’-3’)**
HAGLROS forward	5’- TTTTGCAAAGACAGACGCGG-3’
HAGLROS reverse	5’- TTTAAGGGTGACACTCGGGC-3’
miR-206 forward	5’-GGTGTGTGAAGGAATGTAAGGT-3’
miR-206 reverse	Involved in the kit
HMGA2 forward	5’-ACCCAGGGGAAGACCCAAA-3’
HMGA2 reverse	5’- CCTCTTGGCCGTTTTTCTCCA-3’
U6 forward	5’-GCTCGCTTCGGCAGCACAT-3’
U6 reverse	5’-AAAATATGGAACGCTTCACG-3’
GAPDH forward	5'-GGAGCGAGATCCCTCCAAAAT-3'
GAPDH reverse	5'-GGCTGTTGTCATACTTCTCATGG-3'

### Cell transfection

ShHAGLROS and miR-206 inhibitor were applied to low-express lncRNA HAGLROS and miR-206, while HAGLROS and miR-206 mimics were applied to overexpress HAGLROS and miR-206. Plasmids (GenePharma, Shanghai, China) were applied to transfected into KTC-1 cells using 2μL Lipofectamine 2000 reagent (Invitrogen Life Technologies, USA). Twenty four hours later, cells were harvested and total RNA was extracted using TRIzol reagent and Ambion^®^ DNase I (Invitrogen Life Technologies, USA). To confirm transfection efficiency, qRT-PCR was performed to detect expressions after transfection in each group.

### Luciferase reporter assay

The 3’UTR fragment of HAGLROS was amplified and cloned into the PmeI and XbaI sites of pmirGLO vector (Promega, Madison, WI, USA). The mutant HAGLROS 3’UTR fragment was also generated by site-directed mutagenesis. The constructs were sequenced and named HAGLROS-WT and HAGLROS-MUT plasmids. HMGA2-WT and HMGA2-MUT were also constructed in a similar way. For reporter assays, cell culture was carried out in 24-well plates and then transfected with WT type or MUT type luciferase reporters, and then cotransfected with miR-206 mimics or mimics control. Each assay was repeated three times. After 48 hours following transfection, cells were collected and luciferase activity was detected using the Dual-Glo Luciferase Assay System (Promega, USA) and MicroLumatPlus LB96V luminometer (Berthold, USA). Relative luciferase activity was calculated as ratio of the raw firefly luciferase activity and the renilla luciferase activity.

### Ribonucleoprotein immunoprecipitation (RIP) assay

In short, the cell lysate was blocked with Protein G magnetic beads and incubated with anti-AGO G magnetic beads (Thermo Biotechnology, USA) at 4° C for 1.5 hours. The beads were collected by centrifugation at 700 g for 60S, washed 6 times with RIPA buffer, and resuspended in 50 mmol/L Tris-HCl with a pH of 7.0. Then the magnetic beads were incubated at 70° C for 45 minutes for cross-linking, and then RNA co-IP with anti-AGO antibody was extracted. Finally, the target molecule was quantified by qRT-PCR.

### Clonogenic formation assay

For clonogenic assays, specified numbers of cells which were in logarithmic growth phase were inoculated into 12-well tissue culture plates. Cells treated with HAGLROS, sh-HAGLROS, miR-206 mimics, miR-206 inhibitor and miR-206 mimics + HAGLROS were allowed to adhere for 16 h, and then cells were cultured in incubator (37° C, 5% CO_2_, Saturated humidity). Two to three weeks later, when the colonies were visible, colonies were stained with crystal violet. Colonies of whole well were counted to determine the surviving fraction.

### Flow cytometry analysis

Approximately 48h after transfection with HAGLROS/shHAGLROS/miR-206 mimics/miR-206 inhibitor/HMGA2/shHMGA2/NC, PTC cells were harvested by trypsinization. The cells were then resuspended in PBS and the cells concentration was adjusted to 1 × 10^6^ cells/ml. After double staining with Annexin V-fluorescein isothiocyanate and propidium iodide, cell apoptosis was determined by flow cytometry (BD Biosciences, Franklin Lakes, NJ, USA). All experiments were repeated three times and followed the manufacturer’s guidance.

### Transwell invasion assay

For transwell invasion assays, 2 × 10^5^ transfected cells, which were resuspended in serum-free medium, were plated into the upper chamber of the insert (Corning Costar, Lowell, MA, USA) coated with Matrigel (BD Biosciences, USA). Approximately 600 μL of medium which was replenished with 10% FBS, was injected to the lower chamber. After culture for 48 h, the invading cells were immobilized in 20% methanol and dyed with 0.1% crystal violet. The fixed cells were photographed and counted under an X71 inverted microscope (Olympus, Japan).

### Xenograft tumor model

Healthy BALB/c nude mice (5–6 weeks old, weight 18–20 g) were supplied by the Laboratory Animal Center of Fudan University. These animals were housed and monitored in this center. Experimental procedures and protocols were ratified by Fudan University. Animals were randomized in to 2 groups (5 in each group) and subcutaneously injected with KTC-1 cells, which were transfected with shHAGLROS or shHAGLROS-NC. The tumor size was observed every 5 days after transplantation, using a digital caliper to measure the tumor length (L) and width (W). Volumes of the tumor were calculated following the formula: V (volume) = (L × W^2^)/2. All animals were sacrificed 30 days after injection. The tumor tissues were excised and weighed. A part of the tumor tissues was stored at −80° C for further analysis.

### Fluorescence *in situ* hybridization (FISH) assay

Tissue pretreatment involved removal of tumor tissue samples from the refrigerator, rapid thawing on ice, and rinsing with saline to remove nonspecific cellular components such as blood. After draining excess water with filter paper, the tissue was placed into a 2ml centrifuge tube, 1ml xTissue homogenization buffer was added, and the tumor tissue was broken up using a tissue homogenizer. DNA extraction consisted of the addition of 200μl lysis buffer to the homogenized tissue, mixing with a vortex oscillator, followed by the addition of 200μl wash buffer, mixing with a vortex oscillator and leaving at room temperature for 5 minutes. The samples were centrifuged at 13,000 rpm for 10 min on a benchtop high-speed centrifuge, and the supernatant was discarded, leaving the precipitated DNA. They were washed twice with wash buffer, once more with 70% ethanol, and allowed to dry. Biotin was labeled with biotin, and then fluorescein was labeled with biotin. Finally, avidin was mixed with lncRNA and miRNA probes, and the hybridization reaction was carried out in a constant temperature shaker. The labeled DNA probe solution was mixed with tumor tissue, an appropriate amount of hybridization buffer was added, mixed, transferred to glass slides, and then placed in a constant temperature shaker for hybridization reaction. At the end of hybridization, the slides were washed three times with wash buffer to remove unbound probes. The slides were then sealed with mounting buffer and covered with coverslips.

### Immunofluorescence assay

Tumor tissue samples were selected and pretreated to expose antigens and remove non-specific substances. Secondly, the tissue samples were fixed using paraformaldehyde to maintain the stability of cell morphology and structure, and Triton X-100 was used for permeabilization to promote the binding of antibody to antigen. Third, fluorescent antibodies with strong specificity were selected to avoid high background and unsatisfactory protein localization results. Fourth, the fluorescent antibody (anti-HMGA2, ab207301, Abcam, USA) was added to the tissue sample and incubated at appropriate temperature and humidity for a period of time to make the labeled antibody specifically bind to the antigen. Finally, unbound antibodies were washed with buffer and observed under a fluorescence microscope and the results recorded.

### Statistical analysis

For statistical analysis the GraphPad Prism 6.0 was used (La Jolla, CA, USA). The results were evaluated for statistical significance by the Student’s t-test or the ANOVA test. Error bars represent the S.D. of the mean. A significance level of *P* <0.05 was considered statistically significant, where * denotes a significance level of *P*<0.05, ** denotes a significance level of *P*<0.01, and *** denotes a significance level of *P*<0.001.

### Data availability

The datasets used and/or analyzed during the current study are available from the corresponding author on reasonable request.

## Supplementary Material

Supplementary Figure 1
