# Supplementary material for: LncRNA HAGLROS contribute to papillary thyroid cancer progression by modulating miR-206/HMGA2 expression
Source: Aging (Albany NY). 2023 Dec 18;15(24):14930–44. doi: 10.18632/aging.205321 (PMC10781464; doi:10.18632/aging.205321)
Supplement: Supplementary Figure 1 [file aging-15-205321-s001.pdf]

SUPPLEMENTARY FIGURE

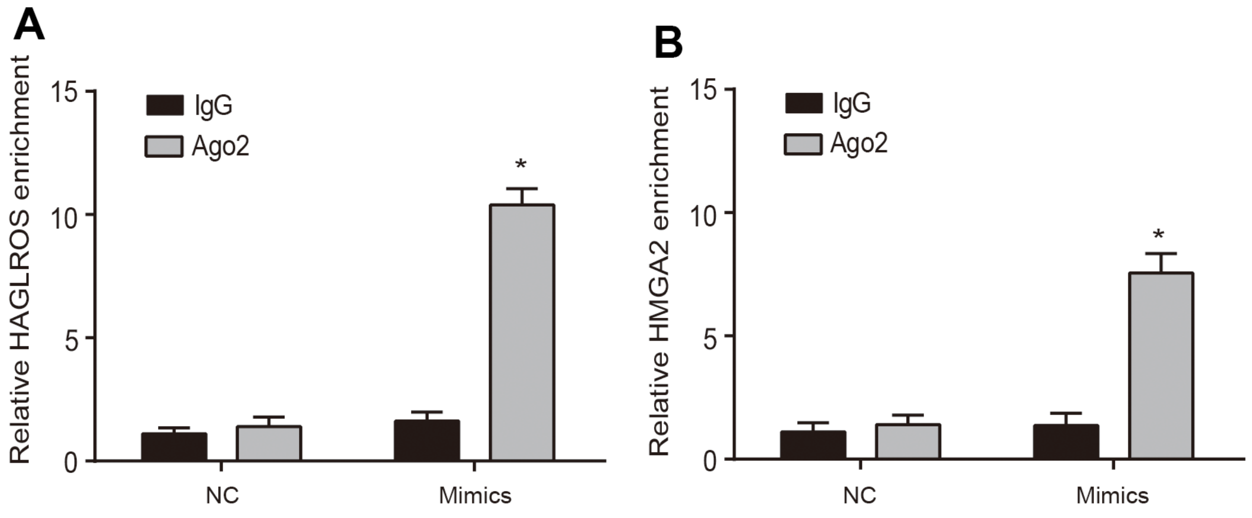

Supplementary Figure 1. The RIP assay was used to detect the binding of HAGLROS, miR-206 and HMGA2. (**A**) The RIP assay was used to detect the binding of HAGLROS and miR-206. (**B**) The RIP assay was used to detect the binding of miR-206 and HMGA2.
